# Authors’ Response to Peer Reviews of “Use of Smartphone Apps for Improving Physical Function Capacity in Cardiac Patient Rehabilitation: Systematic Review”

**DOI:** 10.2196/33179

**Published:** 2021-09-17

**Authors:** Katherine Tuttle, Arpad Kelemen, Yulan Liang

**Affiliations:** 1 Department of Organizational Systems and Adult Health University of Maryland, Baltimore Baltimore, MD United States; 2 Department of Family and Community Health University of Maryland, Baltimore Baltimore, MD United States

**Keywords:** cardiac rehabilitation, physical capacity, exercise, smartphone apps

*This is the authors’ response to peer-review reports for the paper “Use of Smartphone Apps for Improving Physical Function Capacity in Cardiac Patient Rehabilitation: Systematic Review”.*

## Round 1 Review

I. We went through the PRISMA checklist and made changes for better compliance. Some items on the list are not applicable to our article [1].

II. We created the PRISMA diagram as requested by the reviewers.

III. We have done that.

IV. We are fine with transferring to *JMIR Cardio* as suggested by some reviewers.

V. We extended the Abstract as requested.

### Reviewer F [2]

#### General Comments

Thank you for the encouraging comments [2]. We made significant changes in our effort to correct those incorrect statements.

1. We opted to take the sentence out of the Abstract and instead focus more on it within the Introduction. Citations are not typically placed in the Abstract, and cardiac rehabilitation is sometimes covered by insurance plans if eligible. However, not all patients have insurance, so cost can be a deterring factor. This is mentioned as a barrier in the Introduction now.

2. We added this reference [3] and others.

3. Done.

4. Done. We made additional searches as suggested and reported this in the paper.

5. The Forman [4], Layton [5], and Worringham [6] studies were 3 non–randomized controlled trials. We removed them from the Results section. However, they are still mentioned in the Introduction and Discussion sections as support articles, since some vital information was drawn from them.

6. Done.

7. We made these changes as suggested. Depending on location, guidelines and duration can slightly vary. This is now mentioned in the paper.

8. The phase of rehabilitation is included in Table 3. We removed it from the text as requested.

9. We interpret this request by the reviewer to create new numeric codes for the individual outcome measures. Then, we use those codes only in the table and use a legend below the table for the codes. Do we understand it correctly? We slightly disagree with that option; it would make the table itself neat and clean, but it would require a longer time for a reader to read and understand the content and would increase the length of the paper. Nevertheless, we are happy to do this if the reviewer feels strongly about this change. Simply listing the citations after the outcome measures is insufficient, since there are more than 2 options for table cells. However, we significantly simplified Tables 2 and 3. Maybe the current version provides enough simplicity, clarity, and readability for publication.

### Reviewer AI [7]

#### General Comments

1. A complicated problem such as heart failure takes multiple interventions to treat. Although diet does not seem to be directly related to cardiac functional capacity, high sodium diets can aid in retaining fluid in the body, further propagating heart issues, as the heart is too weak to pump the excess fluid. Excess fluid will then push on the chest and sit on the lungs, making exercise difficult, causing shortness of breath while walking, and ultimately making the heart weaker. Multiple interventions are used in treating heart issues, as recommended by the American Heart Association and American College of Cardiology, because it is a complicated organ. Therefore, it is appropriate for smartphone app interventions to include more than one component of cardiac rehabilitation. We added some of this information to the manuscript.

2. Cardiac rehabilitation functional capacity is the primary outcome and was narrowed down to the two main measurements of a 6-minute walk test or peak oxygen uptake in this revised version of our paper. Other outcomes are briefly mentioned in the discussion.

3. We created a PRISMA flow diagram for the study selection process.

4. We deleted the corresponding paragraphs, which were confusing. Also, we simplified the tables to increase their clarity and to better align with our research topic.

## Abbreviations

PRISMA: Preferred Reporting Items for Systematic Reviews and Meta-Analyses
